# 

**Published:** 1894-09-08

**Authors:** 


### CONTENTS.

**Holiday Thoughts for Health Makers** - 457
**Around the Hospitals** - 458
**On Modern Progress in Ophthalmic Medicine and Surgery.** By ROBERT BRUDENELL CARTER, F.R.C.S.—Laminar Cataract (*continued*) - 459
**The Educational Treatment of Idiots, Imbeciles, and Feeble Minded Children.** By G. E. SHUTTLEWORTH, B.A., M.D.—V. Results of Special Training - 460
**Annotations.**—"The Royal and Ancient Game" at St. Andrews—Unlicensed Asylums—The Pauper's Pipe - 461
**Notes and News** - 462
**The Student of Medicine** - 473
**Medical Progress and Hospital Clinics:**
Chronic Catarrh of the Middle Ear.—III. - 463
PROGRESS IN SURGERY.—Bone, Joint, and Orthopædic Surgery - 464
Surgery of the Lungs and Pleura - 466
THE BRITISH MEDICAL ASSOCIATION AT BRISTOL - 427
NEW APPLIANCES AND THINGS MEDICAL - 468
**The Institutional Workshop:**
HOSPITAL ADMINISTRATION.—I. General Hospitals - 469
CANADIAN HOSPITALS. I.—A Visit to the Hospitals at Montreal 470
PRACTICAL DEPARTMENTS - 471
THE STORY OF THE INSANE FROM YEAR TO YEAR - 471
**Scraps and Gleanings** - 472

### EXTRA SUPPLEMENT.—THE NURSING MIRROR.

**News from the Nursing World.**—The Male Nurse—Holidays and the Weather—The Colony of Bethel—The Workhouse Nursing Association—Irish Humour—A Fairy Cake—Nursing at Vancouver—Another Sham Nurse—News from Labrador—Short Items - ccxxvii
**Diet in Disease.**—By Mrs. ERNEST HART. I.—Rickets - cxxix
**The Pride of Mile End** - ccxxx
**Minor Appointments** - ccxxx
**Notes and Queries** - ccxxx
**Character Sketches from a Lunatic Asylum.**—The Patient Whose Language was Unintelligible - ccxxxi
**Everybody's Opinion** - ccxxxii
**Death in Our Ranks** - ccxxxii
**Novelties for Nurses—A Case for Assistance** - ccxxxii

